# Visceral adipose tissue imparts peripheral macrophage influx into the hypothalamus

**DOI:** 10.1186/s12974-021-02183-2

**Published:** 2021-06-21

**Authors:** Kuan-Hui Ethan Chen, Nancy M. Lainez, Meera G. Nair, Djurdjica Coss

**Affiliations:** 1 Division of Biomedical Sciences, School of Medicine, University of California, Riverside, Riverside, CA 92521 USA; 2 Present address: Division of Mathematics and Sciences, Delta State University, Cleveland, MS 38733 USA

**Keywords:** Neuroinflammation, Hypothalamus, Fat transplant, Obesity, Macrophage, Sex differences

## Abstract

**Background:**

Obesity is characterized by a systemic inflammation and hypothalamic neuroinflammation. Systemic inflammation is caused by macrophages that infiltrate obese adipose tissues. We previously demonstrated that high-fat diet (HFD)-fed male mice exhibited peripheral macrophage infiltration into the hypothalamus, in addition to activation of resident microglia. Since this infiltration contributes to neuroinflammation and neuronal impairment, herein we characterize the phenotype and origin of these hypothalamic macrophages in HFD mice.

**Methods:**

C57BL/6J mice were fed HFD (60% kcal from fat) or control diet with matching sucrose levels, for 12–16 weeks. Males and females were analyzed separately to determine sex-specific responses to HFD. Differences in hypothalamic gene expression in HFD-fed male and female mice, compared to their lean controls, in two different areas of the hypothalamus, were determined using the NanoString neuroinflammation panel. Phenotypic changes in macrophages that infiltrated the hypothalamus in HFD-fed mice were determined by analyzing cell surface markers using flow cytometry and compared to changes in macrophages from the adipose tissue and peritoneal cavity. Adipose tissue transplantation was performed to determine the source of hypothalamic macrophages.

**Results:**

We determined that hypothalamic gene expression profiles demonstrate sex-specific and region-specific diet-induced changes. Sex-specific changes included larger changes in males, while region-specific changes included larger changes in the area surrounding the median eminence. Several genes were identified that may provide partial protection to female mice. We also identified diet-induced changes in macrophage migration into the hypothalamus, adipose tissue, and peritoneal cavity, specifically in males. Further, we determined that hypothalamus-infiltrating macrophages express pro-inflammatory markers and markers of metabolically activated macrophages that were identical to markers of adipose tissue macrophages in HFD-fed mice. Employing adipose tissue transplant, we demonstrate that hypothalamic macrophages can originate from the visceral adipose tissue.

**Conclusion:**

HFD-fed males experience higher neuroinflammation than females, likely because they accumulate more visceral fat, which provides a source of pro-inflammatory macrophages that migrate to other tissues, including the hypothalamus. Our findings may explain the male bias for neuroinflammation and the metabolic syndrome. Together, our results demonstrate a new connection between the adipose tissue and the hypothalamus in obesity that contributes to neuroinflammation and hypothalamic pathologies.

## Background

Obesity is of significant public concern. Currently in the USA, over 30% of men and women are classified as obese, with a body mass index (BMI) of ≥30 kg/m^2^ [1]. Obesity is characterized as a chronic inflammatory disorder, suggesting that the immune system responds to high fat diet (HFD) and/or the accumulation of adipose tissue [2–4]. Since obesity is linked to an increased risk of cardiovascular disease, cerebral ischemia, type II diabetes, and reproductive disorders [5, 6], the immune cells that respond to increased adiposity may contribute to these pathophysiological outcomes [7, 8]. Systemic inflammation in obesity, characterized by increased inflammatory cytokines in the circulation, is accompanied by neuroinflammation, specifically in the hypothalamus, characterized by activation of resident immune and glial cells in the brain [4, 7, 9, 10]. The hypothalamus regulates critical functions, such as thermoregulation, circadian rhythm, metabolism, food and water intake, stress, and reproduction. Local inflammation in this area leads to changes in the synaptic connectivity and diminished function of neuronal populations which regulate these physiological processes [9, 11, 12]. We previously demonstrated that, in addition to activation of microglia, which are resident immune cells of the brain, HFD causes an increase in the number of peripheral macrophages in the hypothalamus of obese animals [9]. Our findings are in agreement with other studies that observed an increase in macrophage numbers and suggest that obesity causes infiltration of peripheral immune cells in the hypothalamus [13, 14]. However, others failed to detect infiltration of peripheral immune cells [15] or argued that the increase is attributed to proliferation of perivascular immune cells [16]. To address this discrepancy, here we perform additional analyses to investigate profiles and origin of hypothalamic macrophages in obesity.

In obesity, neuroinflammation and macrophage infiltration occur in specific regions of the hypothalamus [9]. Macrophage infiltration may be facilitated by the increased permeability of the blood-brain barrier or elicited by active recruitment, via chemokines. Increased permeability in obesity may occur due to downregulation of several tight junction proteins [17–19]. However, reasons for regional differences are not clear. The hypothalamus is bordered by several areas that contain fenestrated capillaries characterized by a leaky blood-brain barrier [20]. Anteriorly, the preoptic area (POA) surrounds the organum vasculosum laminae terminalis (OVLT), and basally, the arcuate nucleus (ARC) is juxtaposed to the median eminence (ME), where secretion of the pituitary-regulating neuropeptides occurs from neuronal terminals. Both OVLT and ME contain fenestrated capillaries, which allow surrounding hypothalamic nuclei to sense the changes in the periphery [21, 22]. It is still not clear if obesity-mediated neuroinflammation in these areas specifically occurs because metabolic signals influence hypothalamic cells, or because the fenestrated capillaries allow infiltration of peripheral immune cells.

Increase in adipose tissue size correlates with macrophage infiltration into the fat depots and pro-inflammatory cytokine production in both humans and mice [23–25]. Obese adipose tissues produce monocyte chemoattractant protein-1 (MCP-1, or CCL2 chemokine), a ligand for CCR2, which recruits monocytes and leads to macrophage activation [26, 27]. We and others demonstrated that leptin is a potent chemoattractant for macrophages, and the increased leptin in obesity may contribute to accumulation of macrophages in the adipose tissues [28–30]. A role for CCR5, and its ligand RANTES, has been suggested to play a role in macrophage recruitment to adipose tissues and their shift to a pro-inflammatory profile [31]. Yet, others suggest that adipose tissue secretion of CXCL14 is critical for macrophage accumulation [32]. CXCL14 is an important modulator of immune cell migration [33], since it competes with CXCL12 (SDF-1, stromal derived factor 1) for binding to CXCR4 [34]. This allows CXCR4, which has an important role in angiogenesis, stem cell and neuronal migration, and neuroinflammation [35, 36], to toggle between immune cell trafficking and retention. Herein, we analyzed these chemokine receptors in macrophages recruited to the hypothalamus.

Macrophages, which infiltrate several tissues following activation by increased adiposity, contribute to obesity-mediated pathologies [3, 8]. Peripheral monocytes infiltrate the liver and give rise to proinflammatory macrophages, contributing to non-alcoholic fatty liver disease (NAFLD) [37, 38]. Macrophages also infiltrate the muscle of mice and humans, where they contribute to insulin resistance [39, 40]. We observed macrophage recruitment to the hypothalamus in obese animals, which caused hypothalamic neuroinflammation [9]. Increased inflammation in the central nervous system (CNS) of obese individuals may contribute to neuropathologies, such as cerebral ischemia and dementia [41–43]. Therefore, further investigation into macrophage infiltration in the hypothalamus may elucidate their contribution to neuroinflammatory diseases.

There are profound sex differences in adiposity and obesity-associated diseases [44]. In men, obesity is associated with heart disease and myocardial infarction, while obese women are more prone to ischemic stroke [45]. Differential accumulation of adipose tissue may contribute to sex differences in obesity-associated diseases [46], but mechanisms that contribute to higher risk for diseases, such as heart disease and metabolic syndrome in men, are not clear [45]. Our previous study determined that major obesity-mediated sex differences in inflammatory and endocrine changes occurred irrespective of ovarian estrogen [9] and were due to intrinsic differences in the macrophages [28–30]. While it is known that males on HFD gain more visceral adipose tissue than females [46], that recruits and activates macrophages [23, 47], the reasons for these differences are only beginning to emerge. Previous studies analyzing obesity-mediated inflammation concentrated on male mice only. To address this gap, we analyzed both males and females to determine sex-specific responses to diet-induced obesity.

Our study presented here demonstrates increased expression of the neuroinflammatory signature genes in the hypothalami of HFD-fed animals, using NanoString neuroinflammation panel, in both region- and sex-specific manner. To determine the major players in hypothalamic neuroinflammation, we performed flow cytometry to distinguish peripheral macrophages, which are CD45^high^, from resident microglia, which are CD45^low^, and analyzed their numbers and phenotypes in the hypothalamus. We also compared hypothalamic macrophages to macrophages in the adipose tissues and peritoneal cavity. Last, we performed fat transplant studies to investigate sources of macrophages in the hypothalamus. We determined that macrophages that infiltrate the hypothalamus display a pro-inflammatory phenotype and originate from visceral adipose tissue. Together, our results reveal a previously unknown connection between the adipose tissue and the brain, whereby HFD-activated macrophages from the visceral fat pad infiltrate the hypothalamus in male mice. These results suggest that the function of macrophages in the hypothalamus of obese mice is determined by their origin from the adipose tissue and has functional outcomes for obesity-induced neuroinflammation.

## Materials and methods

### Animals

All experiments were performed with approval from the University of California (Riverside, CA) Animal Care and Use Committee and in accordance with the National Institutes of Health Animal care and Use Guidelines. C57BL/6J mice were maintained under a 12-h light, 12-h dark cycle and received food and water ad libitum. C57BL/6J male and female mice were obtained from Jackson labs at weaning age and after a week acclimatization on normal chow, placed on either a high fat diet (HFD, D12492, 60% kcal from fat; 5.21 kcal/g; carbohydrate 20% kcal, protein 20% kcal, fat 60% kcal (lard 0.32 g/g diet, soybean oil 0.03 g/g); Research Diet, New Brunswick, NJ) or control diet (CTR, D12450J, 10% kcal from fat; matching sucrose levels to HFD; 3.82 kcal/g; carbohydrate 70% kcal, protein 20% kcal, fat 10% kcal (lard 0.02 g/g diet, soybean oil 0.025 g/g); Research Diet, New Brunswick, NJ) for 12–20 weeks, as indicated for each experiment.

#### Estrous cyclicity

Female mice were assessed for estrous cycle stage with daily vaginal smears. Animals were handled daily, at the same time of day, to account for any potential stress that handling may cause. Vaginal lavage was performed daily (between 9 and 10 am) by flushing the vagina with distilled H_2_O. Collected smears were mounted on glass slides and examined microscopically for cell types. Estrous cycle stages determined during the first week of vaginal smearing were not deemed reliable due to acclimatization. During the second week of vaginal smearing, tissue samples for subsequent studies were collected in diestrus identified between 9 and 11 am.

### NanoString analysis of hypothalamic gene expression

After diet, mice were perfused with ice cold PBS, and the brains rapidly removed and flash frozen in isopentane on dry ice. Coronal brain sections of 300 um were obtained using cryostat and two regions analyzed for gene expression changes by Nanostring. Medial ventral 1mm^2^ was dissected from the section that started at Bregma 0.86, according to Franklin and Paxinos mouse brain atlas, and contained POA (preoptic area) and OVLT (organum vasculosum laminae terminalis). Additionally, medial ventral 1mm^2^ section was dissected from a 300-um section starting at Bregma −1.58 that contained ARC (arcuate nucleus) and ME (median eminence). RNA was isolated using the RNAqueous®-Micro Kit (Ambion) and quantified using Nanodrop. Gene expression in 50 ng RNA per sample was analyzed using the NanoString instrument, according to manufacturer’s instruction, with the nCounter Mouse neuroinflammation Panel (770 genes, gene list available at the manufacturer’s website). The panel was customized with an addition of 30 custom probes for hypothalamic neuropeptides and their receptors. Only samples with an RNA integrity number RIN > 7 were used for Nanostring. All data passed QC, with no imaging, binding, positive control, or CodeSet content normalization flags. The analysis of data was performed using nSolver Analysis Software 4.0, including nCounter Advanced Analysis (version 2.0.115). Genes with the expression lower than the limit of detection after background subtraction, and compared to negative controls included in the panel, were excluded. Seven housekeeping control genes that are included in the panel were used for normalization. Results are plotted as log fold change vs. log p value, and genes with changes higher than 25% were indicated with colors in the figures: red indicates genes induced by HFD and green genes repressed by HFD.

### Fat transplant

The adipose tissue was transplanted as described previously [48–50]. Briefly, male donor mice (C57Bl/6J CD45.2 from Jackson labs) were fed control or high fat diet consistent with description above. After 12 weeks on respective diets, animals were euthanized and intra-abdominal perigonadal (epididymal) visceral fat depots were isolated and kept in saline at 37°C for the maximum of 30 min until transplantation. These depots from both sides were transplanted into the visceral fat pad of mature recipient male mice (C57Bl/6 CD45.1). Since each donor mouse had left and right side fat pad, one fat pad was transplanted into the mouse that was placed on control diet and the other into the mouse that received HFD to further control for potential individual differences that may arise. We used congenic CD45.1 and CD45.2 on the C57BL/6J genetic background, which only differ in the CD45 allele, minimizing problems that may arise with transplantation. This allows us to distinguish between CD45.1 recipient cells and CD45.2 donor cells. Recipient mice were anesthetized with 2% isoflurane using vaporizer and sterile procedure. Donor slices of fat were transplanted into the visceral fat pad as described before. Briefly, endogenous epididymal fat pad of the recipient was sliced with a scalpel and donor fat was carefully lodged deep between the folds of endogenous fat pad. Animals were monitored for 48 h recovery and then placed on the control or HFD for an additional 7 weeks.

### Histological analyses and immunohistochemistry

At the conclusion of diet exposure, mice were anesthetized and perfused with 20 ml PBS and 20 ml 4% paraformaldehyde; and tissues collected. Adipose tissue depot that contained transplanted fat as well endogenous fat was either embedded in paraffin, or frozen in OCT. Paraffin blocks were cut to 20 μm, deparaffinized in xylene and rehydrated, and H&E staining performed to assess morphology. Frozen tissue was cut to 20-μm sections using Leica cryostat. Slides were blocked with 20% goat serum and incubated with primary antibodies against CD31 (1:5000, 553370, BD Bioscience, San Jose, CA), at 4°C for 48 h. After PBS washes, slides were incubated with FITC/Alexa 488 goat IgG (1:300, Molecular Probes, Eugene, OR) for 30 min. Secondary antibody-only controls were performed to determine antibody specificity. Images were obtained using Leica microscope.

### Flow cytometry

Antibodies used for flow cytometry are listed below. Tissues from each mouse were processed separately as part of a 3–4-mouse cohort per group, with each experiment repeated 3 times. In brief, mice were perfused with ice cold PBS, and the adipose tissue was collected from gonadal fat pads, rinsed in cold PBS, weighed, minced with razor blade, and digested enzymatically with 3 mg/mL collagenase at 37 °C for 1 h. Suspension was passed through 70-um cell strainer, centrifuged to pellet stromal vascular fraction, cells collected, and 2 million cells labeled for flow cytometry analyses. Cells were Fc-blocked with anti-CD16/CD32 (1:100, 553141, BD Biosciences, San Jose, CA) followed by surface marker staining with antibodies to F4/80 (Brilliant Violet 650; 1:400, 123149, BioLegend, San Diego, CA) and CD11b (Brilliant Violet 605; 1:400, BD Biosciences, San Jose). Flow analysis was performed with BD LSR II Flow Cytometer using the following gating strategy: cells, singlets, live cells, and F4/80+CD11b+.

Hypothalami were processed as described before [9]. Briefly, hypothalami cell suspensions were generated by enzymatic dissociation using collagenase and dispase and applied to a discontinuous 60%/30% percoll gradient. Cells were collected from the interface, blocked with anti-CD16/CD32 (1:100, 553141, BD Biosciences, San Jose, CA), and incubated with anti-CD45 APC-eFluor® 780 (1:300, 47-0451, eBioscience, San Diego, CA) and anti-CD11b Brilliant Violet 605 (1:300, 563015, BD Biosciences, San Jose, CA) in PBS, 5% EDTA, and 0.4% BSA. Flow analysis performed using BD LSR II Flow Cytometer using the following gating strategy: cells, singlets, live cells, and CD45+CD11b+.

Peritoneal cavity cells (PEC) were collected following injection of 10-ml ice-cold PBS into the peritoneum. Cells were then blocked with anti-CD16/CD32 (1:100, 553141, BD Biosciences, San Jose, CA), and macrophages were identified using anti-F4/80 Brilliant Violet 650 (1:400, 123149, BioLegend, San Diego, CA) and anti-CD11b (N418; Brilliant Violet 605; 1:400, BD Biosciences, San Jose) using the following gating strategy: cells, singlets, live cells, and F4/80+CD11b+.

For cell surface molecules, the following antibodies were used: CD206 PE (1:400, 12-2061-82, eBioscience, San Diego, CA), CD11c Brilliant Violet 510 (1:400, 117337, BioLegend, San Diego, CA), CD36 PE/CY7 (1:400, 102615, BioLegend, San Jose, CA) or CD36 APC (1:400, 17-0362-82, eBioscience, San Diego, CA), PDL1 PE/Dazzle 594 (1:400, 124323, BioLegend, San Diego, CA), CCR2 Alexa Fluor 647 (1:400, 150603, BioLegend, San Diego, CA), CCR5 PE (1:400, 12-1951-82, ThermoFisher, Chino, CA), CXCR4 Brilliant Violet 421 (1:400, 146511, BioLegend, San Diego, CA), CD45.1 APC-780 (1:400, 47-0453-82, eBioscience, San Diego, CA), and CD45.2 PE-CY7 (1:400, 25-0454-82, eBioscience, San Diego, CA). Flow analysis was performed using BD LSR II Flow Cytometer. Results were analyzed using FlowJo software (Tree Star, Inc.), and statistical differences were determined by Student’s T test and Tukey’s post hoc test using Prism GraphPad software.

### Statistical analyses

We focused on differences between control and HFD within males and females. Statistical differences (*p* < 0.05) were determined by Student’s t test or ANOVA where appropriate followed by Tukey’s test for multiple comparisons using Prism software (GraphPad, CA). When an interaction between cohorts and diets were analyzed a two-way ANOVA was conducted by Prism software.

## Results

Initial reports of hypothalamic neuroinflammation in obesity postulated that neuroinflammation is caused by stress and dysfunction of the feeding circuitry neurons, anorexigenic POMC, and orexigenic NPY, in the arcuate nucleus (ARC), since inflammation was detected specifically in this region [11]. We detected neuroinflammation in the preoptic area (POA) surrounding the OVLT, in addition to the ARC and median eminence [9]. Thus, we postulated that inflammation occurs due to the proximity to the fenestrated capillaries. To investigate this hypothesis, we compared gene expression profile in mice fed control or high fat diet (HFD) using NanoString neuroinflammation panel. We analyzed males and females separately, since females exhibited less neuroinflammatory changes when exposed to the HFD in our previous studies [9, 28]. Females were estrous cycle staged and analyzed in diestrus, to minimize changes in steroid hormone levels. Male mice after 14-week HFD weighed ~175% of males fed control (Ctr) diet (48 g compared to 27.5, respectively). Although female mice gain less weight than male mice, when compared to the Ctr fed mice, HFD female mice weighed ~140% of Ctr fed females (21.2 g Ctr, and 30 g HFD, data not shown).

**Fig. 1:**
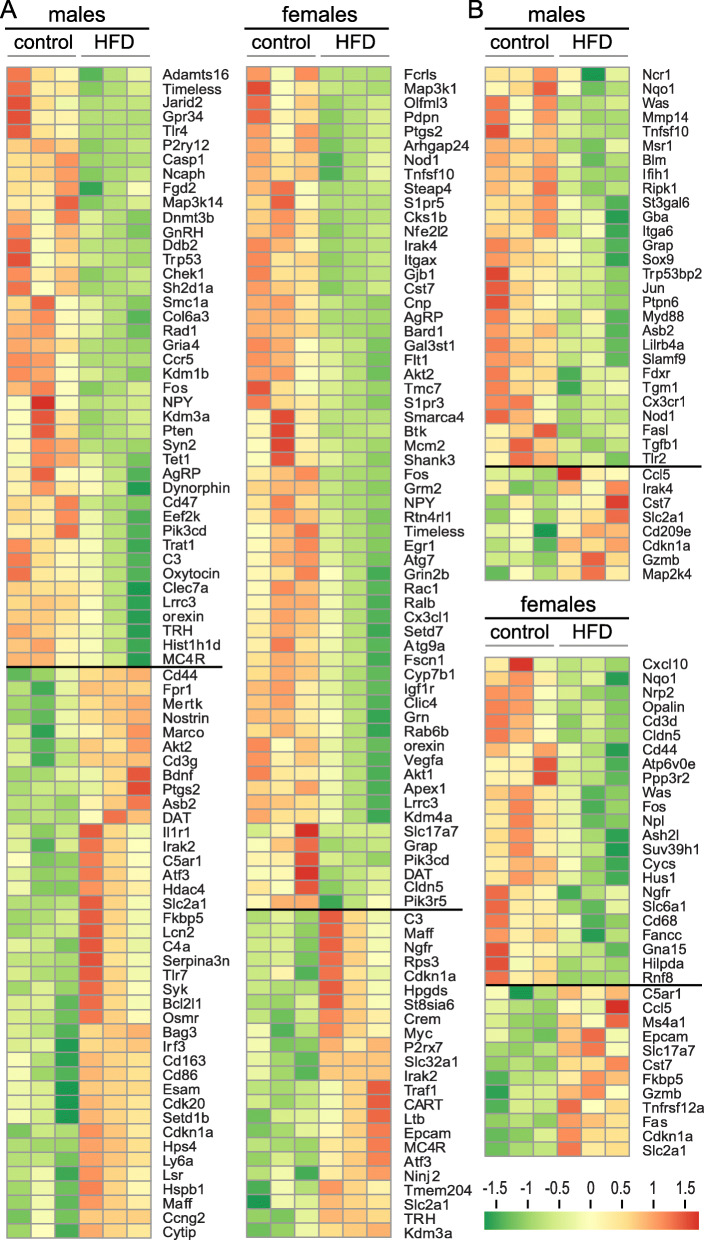
NanoString analysis demonstrates significant changes in gene expression with HFD. Male and female mice were fed control (Ctr) or high fat diet (HFD) for 14 weeks. **A** Hypothalami from 3 mice per group were dissected and the area containing the arcuate nucleus, and the median eminence from the 300-um coronal section was excised and 50 ng RNA used in NanoString analysis. Genes exhibiting significant changes in expression are highlighted with heat-maps. **B** The anterior portion containing POA and OVLT was excised and used in a separate NanoString neuroinflammation panel. Genes exhibiting significant changes in expression are listed

We analyzed gene expression changes in the 300-um coronal posterior section of the hypothalamus that contained ARC and median eminence, and in the 300-um coronal anterior section from Bregma 0.86 that contained POA and OVLT. The complete lists of genes that change in males and females, in the posterior section and in the anterior section are presented with heatmaps in Fig. 1A and B, respectively. In the male posterior ARC-containing section, 82 genes exhibited significant changes, out of 770 genes in the neuroinflammation panel, and 30 additional genes in the custom panel that are important for hypothalamic function (Fig. 1A). Forty-two genes were downregulated, while 40 genes were upregulated, delineated with a line. In the female posterior portion, also 82 genes changed expression, of which 59 were downregulated and 23 upregulated. In the anterior POA-containing section, 36 genes changed in males, of which 28 genes were downregulated and 8 genes were upregulated. In females, 35 genes total changed, 23 downregulated, and 12 upregulated (Fig. 1B). Thus, posterior sections from males and females exhibited a larger number of genes that changed significantly with HFD than anterior sections, which may correlate with the larger permeability of fenestrated capillaries in the median eminence than in OVLT [51, 52].

**Fig. 2:**
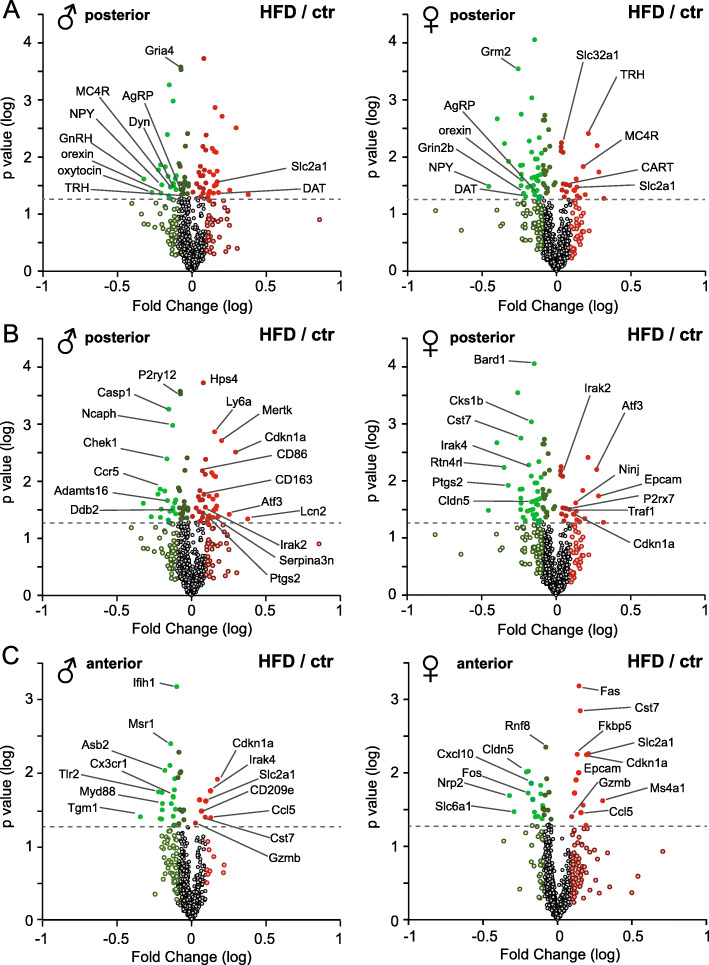
Gene expression profile indicates neuroinflammatory changed in mice fed HFD. **A** A custom panel of 30 genes critical for hypothalamic function was included and several genes highlighted. **B**, **C** Highly regulated genes from the 770 gene off-the-shelf neuroinflammation panel are highlighted from the posterior sections (**B**) and anterior sections (**C**). The panel contains 7 housekeeping genes used for normalization and 8 genes to determine lower limit. The data analysis was performed using nSolver Analysis Software 4.0 and nCounter Advanced Analysis. Data were plotted as log fold change on x-axis vs. log p value on y-axis. Red indicates genes that increase with HFD compared to control fed mice, while green indicates genes that decease with HFD compared to controls

Changes in expression of neuropeptides and receptors that mediate reproduction and metabolic function are highlighted in Fig. 2A. Not surprisingly, mRNA levels for orexigenic neuropeptides, orexin, NPY, and AgRP were decreased in HFD-fed males and HFD-fed females compared to lean mice. Slc2a1 gene that codes for GLUT1, the ubiquitous high-affinity glucose transporter that is the major glucose transporter across the blood-brain barrier, was upregulated in both HFD-fed males and females, in the posterior ARC-containing section and the anterior POA-containing section (Fig. 2A and C). The majority of genes were differentially regulated in males and females with HFD. Of interest, Gria4 gene for AMPA 4/GluR4 glutamate receptor was repressed specifically in HFD-fed males, whereas Grm2 coding for mGLUR2 metabotropic glutamate receptor 2, and Grin2b coding for NMDA receptor 2B, were repressed specifically in HFD-fed females. As we demonstrated before [9, 53] and consistent with previous studies [54, 55], GnRH (gonadotropin-releasing hormone) gene, critical for reproductive function, was repressed specifically in HFD-fed males. TRH (thyrotropin-releasing hormone), which causes increased thyroid hormone levels leading to a higher metabolic rate [56], was upregulated only in HFD-fed females. Although leptin upregulates TRH expression [57], sex differences were not reported before and our results point to significant sex differences in TRH expression in obesity. In response to the HFD, females also upregulated MC4R, melanocortin 4 receptor that regulates food intake and energy expenditure [58]. This increase was specific for HFD-fed females, although both males and females downregulated AgRP, which can bind MC4R [58]. Our results indicate that following HFD, only females exhibit changes in genes that may increase metabolic rate and energy expenditure. These differences may play a role in female protection from some obesity-induced pathologies.

Genes that play a role in neuroinflammation are highlighted in 2B (posterior ARC-containing hypothalamic section) and 2C (anterior POA-containing hypothalamic section). Consistent with obesity being a low grade chronic inflammatory disorder, both the anterior and the posterior areas of the hypothalamus exhibited increase in neuroinflammatory signature genes. Cdkn1a, that encodes p21 cyclin-dependent kinase inhibitor 1A [59], was upregulated in both HFD-fed males and HFD-fed females compared to lean mice in the posterior and anterior section. Many genes demonstrated a sex-specific or region-specific pattern. Males exhibited a larger number of pro-inflammatory genes that are increased with HFD, correlating with higher neuroinflammation in obese male mice [9]. For example, HFD males, especially in the posterior section (Fig. 2B), demonstrated induction of Ptgs2, prostaglandin-endoperoxide synthase = COX2, MerTK that is the involved in phagocytosis by macrophages [60], CD86 and CD163. Genes that are characteristic of reactive astrocytes, such as lipocalin-2 (Lcn2) and Serpina3n [61], were increased specifically in the posterior portion in HFD-fed males compared to lean males. Females, however, showed changes in endothelial cell specific genes, in the ARC (Fig. 2B) and in the POA (Fig. 2C): Epcam, epithelial cell adhesion molecule, was increased in HFD, while Cldn5, claudin 5 that regulates tight junctions, was repressed in HFD. Some region-specific genes were also identified, such as stress-induced transcription factor Atf3 [62] that was upregulated in both HFD-fed males and females in the ARC; while Gzmb, granzyme B serine protease involved in chronic inflammation, and Ccl5, RANTES chemokine, were upregulated specifically in the POA of both HFD-fed males and females. Therefore, the current work established neuroinflammation gene expression signature associated with HFD and demonstrated sex and region dependent differences.

### Hypothalamic macrophages share the same surface markers as adipose tissue macrophages (ATMs)

**Fig. 3:**
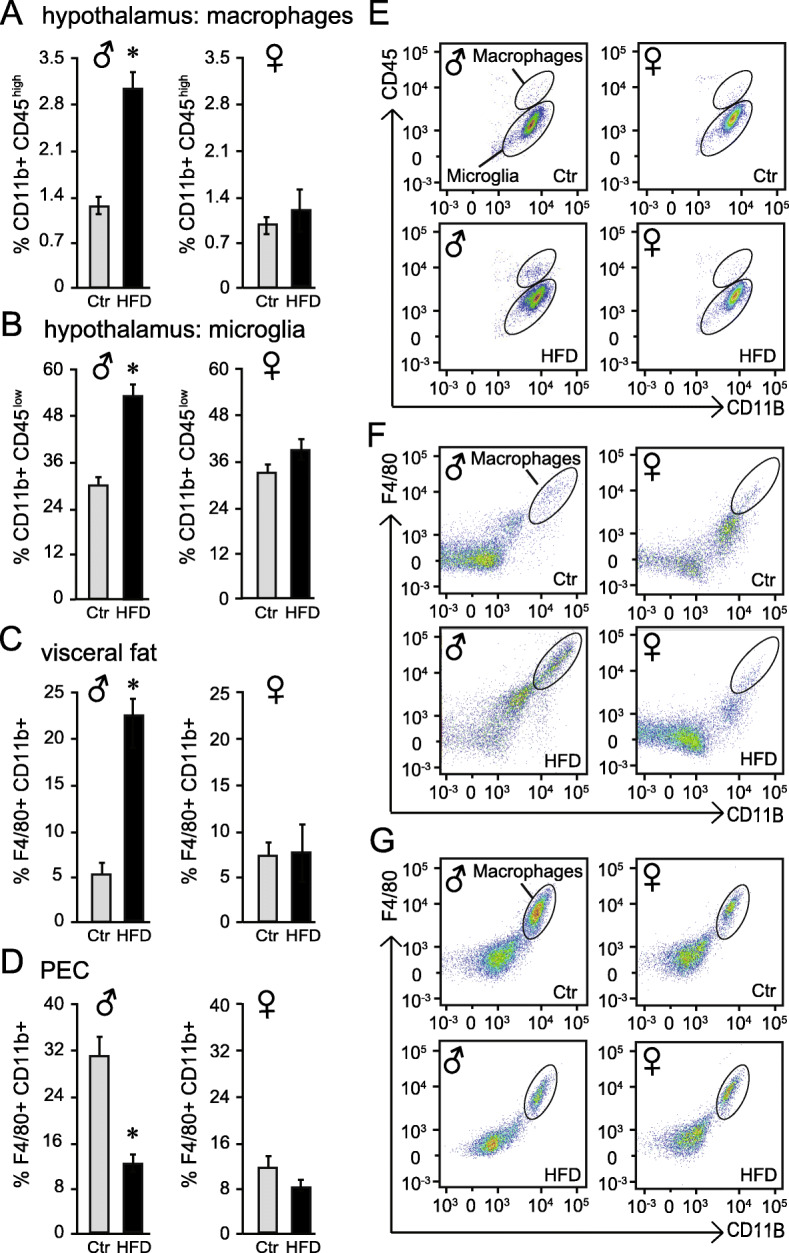
Flow cytometry analyses of the hypothalamus (**A**, **B**, **E**), visceral fat pad (**C**, **F**), and peritoneal cavity (PEC, **D**, **G**) demonstrated macrophage migration following HFD. **E** An example of flow cytometry gating, indicating the presence of the CD45^high^ macrophage population that can be distinguished from the CD45^low^ microglia specifically in the hypothalami of HFD male mice, but not in female mice. These populations were quantified and presented as the % of all live cells in A (macrophages, CD11b+CD45^high^) and B (microglia, CD11b+Cd45^low^). **C** Quantification of F4/80+CD11b+ macrophages in the stromal vascular fraction of visceral adipose tissue following Ctr and HFD in males (left) and females (right). **F** An example of the flow cytometry gating strategy from visceral fat. **D**, **G** Peritoneal cavity (PEC) cells were analyzed by flow cytometry (gating example **G**) and the percent of F4/80+CD11b+ macrophages of all live cells are presented graphically in **D**, males on the left and females on the right. n=9–12 total, 3 repeats consisting of 3–4 mice per group. Statistical significance (*) between Ctr (gray bars) and HFD (black bars) were determined by Student’s T test followed by Tukey’s post hoc test

Our previous study reported activation of microglia, resident immune cells in the brain, and infiltration of peripheral macrophages into the hypothalamus specifically in male mice [9]. Here, we compare and contrast macrophage influx in the hypothalamus and in the adipose tissue in male and female mice following HFD, using flow cytometry. Microglia and macrophages can be distinguished from other brain cells due to the presence of CD11b, and from each other by their differential expression of CD45, since microglia are CD11b+ CD45^low^ and macrophages are CD11b+ CD45^high^ [63, 64] (Fig. 3E, examples of gating in the brain). HFD caused an increase in macrophages from 1.3 to 2.9% of all hypothalamic cells in male mice (Fig. 3A). In the female hypothalami, the percent of macrophages was unchanged with diet. Males on HFD also exhibited an increase in microglia from 31 to 52% (Fig. 3B). In females, the percentages of microglia were the same irrespective of diet (Fig. 3B, right), consistent with a lack of morphological changes in female microglia that we reported before [9].

In agreement with previously published reports, we detected macrophage accumulation in the adipose tissue of HFD male mice. We analyzed double positive F4/80+CD11b+ population in both males and females using flow cytometry (Fig. 3F, example of gating). Proportion of macrophages in male visceral adipose tissue increased from 5.3% in Ctr lean male mice to 22.8% in HFD obese male mice (Fig. 3C). In the female visceral adipose tissues, macrophages comprised 7.6% in Ctr animals and 7.8% in HFD-fed animals. Thus, female mice were resistant to macrophage accumulation, despite weight gain.

Furthermore, we analyzed peritoneal cavity (PEC) cells (example of gating strategy Fig. 3G) and determined that the proportion of macrophages (F4/80+CD11b+) decreased from 31% in Ctr male mice to 13.7% in HFD male mice (Fig. 3D, left). In the female PEC, macrophages comprised 10.9% in Ctr animals and 8.4% in HFD animals, which was not a significant change (Fig. 3D, right). The results from male mice are consistent with previous reports demonstrating that after infection-induced inflammation, the percentage of F4/80+CD11b+ cells decreases in PEC, in the little understood, the so-called macrophage disappearance reaction [65–67]. Taken together, we determined that diet-induced obesity causes an increase in the proportion of microglia, and influx of macrophages in the hypothalamus and into the adipose tissue in male mice, but decrease in the PEC.

**Fig. 4:**
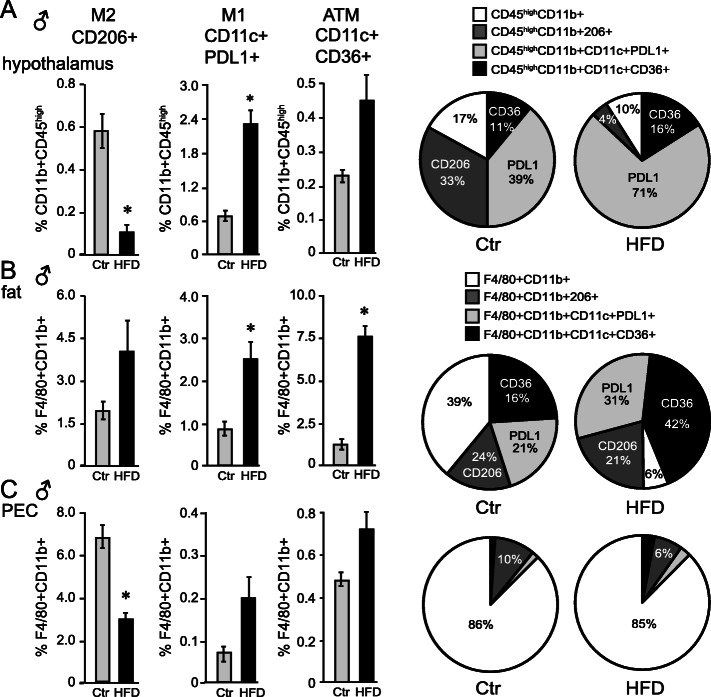
Peripheral macrophages that increase in the hypothalamus following HFD express pro-inflammatory markers. **A** The percent of hypothalamic macrophages defined as CD11b+CD45^high^ that express M2-like marker CD206, M1-like pro-inflammatory marker CD11c+PDL1+, and the marker of adipose tissue, metabolically activated, macrophages (ATM) CD11c+CD36+. Bar graphs present the % of all live cells, while pie charts present their proportion of only macrophages. **B**, **C** Visceral fat depot (**B**) or PEC (**C**) macrophages defined as F4/80+CD11b+ that contain CD206+, or CD11c+PDL1+, or CD11c+CD36+. Bar graphs represent the % of macrophages with these markers of all live cells, while pie charts present the macrophage populations only. n=9–12 per group. Statistical significance (*) between Ctr (gray bars) and HFD (black bars) were determined by Student’s T test followed by Tukey’s post hoc test

Since females, despite increased adiposity and weight gain, lacked changes in macrophage numbers in any of the tissues we analyzed, our subsequent studies concentrated on males, to determine changes in macrophage phenotype. To assess the macrophage phenotype in the hypothalamus, the adipose tissue, and the PEC of male mice, we used traditional markers for macrophage polarization; pro-inflammatory M1-like macrophages, characterized as CD11c+, or anti-inflammatory M2-like macrophages, characterized as CD206+ (Fig. 4). Additionally, macrophages present in the adipose tissues are characterized by the expression of CD36, which binds oxidized low-density lipoprotein, phospholipids, and long-chain fatty acids and are referred to as metabolically activated macrophages or adipose tissue macrophages (ATMs) [68–70]. Thus, ATMs are F4/80+CD11b+CD11c+CD36+. To distinguish ATMs from other pro-inflammatory, CD11c+ macrophages (F4/80+CD11b+), M1 marker, PDL1+ (programmed death-ligand 1) was included [71]. Based on these markers, we analyzed the phenotype of male macrophages that accumulated in the hypothalamus and were associated with neuroinflammation in males.

In the hypothalami, HFD resulted in a decreased proportion of CD206+ M2-like macrophages from 0.58% of all cells to 0.13% (Fig. 4A). The proportion of CD11c+PDL1+ M1 pro-inflammatory macrophages increased from 0.7 to 2.3%, whereas the percent of CD11c+CD36+ ATM increased from 0.23 to 0.45%, which was not significant. Pie-charts indicate the proportion of different macrophage populations and demonstrate that the major shift in macrophage phenotype in the hypothalamus in obesity was an increase of the PDL1+ M1 population (from 39 to 71%) and a decrease of the CD206+ M2 population (from 33 to 4%; Fig. 4A).

Adipose tissue macrophages (F4/80+CD11b+) showed an increase in the proportion of CD11c+ cells with HFD: CD11c+PDL1+ M1 increased from 0.7 to 2.46%, and CD11c+CD36+ ATM increased from 1.3 to 7.5% (Fig. 4B). To illustrate these changes within the macrophage population only, pie charts also showed that both F4/80+CD11b+CD11c+PDL1+ M1-like and F4/80+CD11b+CD11c+CD36+ ATM macrophage populations increased (from 21 to 31% and from 16 to 42%, respectively), while the proportion of other F4/80+CD11b+ cells decreased (from 39 to 6%). The PEC macrophages showed a decrease in the proportion of CD206+ M2-like macrophages with HFD from 6.7 to 3%, while CD11c+ M1-like macrophages did not change (Fig. 4C). Not surprisingly, the pie charts demonstrated that the vast majority of F4/80+CD11b+ macrophages in PEC did not express any of these markers (white) and that the M2-like CD206+ population decreases from 10 to 6% with HFD. Therefore, in response to HFD, male mice increase the population of pro-inflammatory CD11c+ macrophages, both CD36+ ATM and PDL1+ cells, in the adipose tissue. In the hypothalami, M1-like pro-inflammatory macrophages PDL1+ increase, while M2-like CD206+ macrophages decrease with HFD. This implied there was either potential infiltration of M1 pro-inflammatory macrophages into the hypothalamus or putative conversion of other subtypes of recruited or resident macrophages into M1-like cells in HFD-male mice.

### Obesity alters chemokine receptor levels in macrophages at distinct sites

**Fig. 5:**
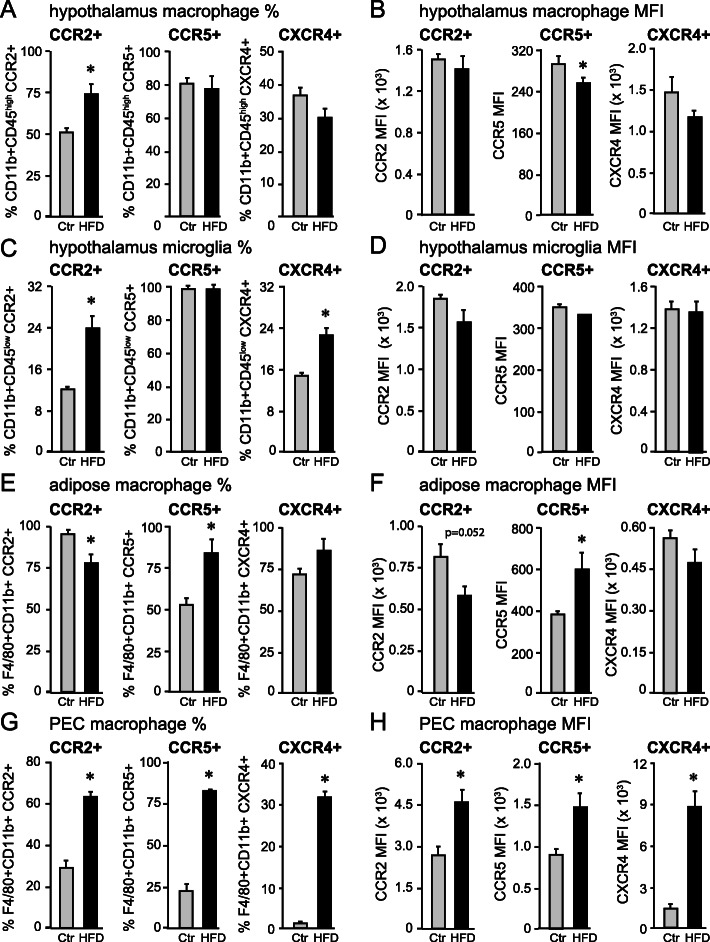
Chemokine receptor levels. **A**, **C**, **E**, and **G** The proportion of macrophages (**A**, **E**, **G**)/microglia (**C**) that express CCR2, CCR5, or CXCR4 receptors. **B**, **D**, **F**, and **H** Mean fluorescent intensity (MFI) indicates the levels of expression of CCR2, CCR5, or CXCR4 in macrophages. **A**, **B** Hypothalamic macrophages CD11b+CD45^high^; **C**, **D** hypothalamic microglia CD11b+CD45^low^; **E**, **F** visceral fat macrophages F4/80+CD11b+; **G, H** Peritoneal cavity (PEC) macrophages F4/80+CD11b+. The proportion of CCR2-positive cells increases in the hypothalamic macrophages and microglia, while the proportion of CCR5+ macrophages increases in the adipose tissue. PEC macrophages increase proportions of CCR2-, CCR5-, and CXCR4-positive cells. Statistical significance (*) between Ctr (gray bars,) and HFD (black bars) were determined by Student’s T test followed by Tukey’s post hoc test

CCL2, CCL5 (RANTES), and CXCL14 may serve as chemoattractants for macrophages in HFD-fed male mice, as discussed above. We analyzed the expression of their receptors, CCR2, CCR5, and CXCR4, respectively, in macrophages from the hypothalamus, adipose tissue, and PEC. In the hypothalamus, the proportion of CD45^high^CD11b+ that express CCR2 increased from 50 to 72% with HFD, while the proportion of CCR5+ and CXCR4+ macrophages did not change (Fig. 5A). Next, surface levels of these receptors in each cell were determined by mean fluorescent intensity (MFI). In the hypothalamus, only CCR5 decreased slightly, while CCR2 and CXCR4 levels did not change (Fig. 5B). Since we distinguished microglia as CD45^low^CD11b+, we determined the proportion of microglia that express these receptors. CCR2-expressing microglia number almost doubled in HFD, from 12% in Ctr to 23% in HFD (Fig. 5C). Our studies agreed that most microglia, 99%, normally express CCR5 [72], which did not change with HFD. Interestingly, proportion of microglia that express CXCR4 increased from 14 to 21% in HFD (Fig. 5C), which may confirm a role of CXCR4 in microglia surveillance [73] and brain plasticity [36]. None of the levels of these receptors changed with HFD, determined by MFI (Fig. 5D). Therefore, these results indicate the proportion of cells that express these receptors changes, but not the levels of expression within the cells.

In the adipose tissue, macrophages that express CCR2 decreased from 95% in Ctr to 75% in HFD (Fig. 5E), and we postulate, given that CCL2 may have served as a chemoattractant to adipose tissue, its receptor is downregulated once macrophages infiltrate the tissue. Macrophages that express CCR5+ increased from 53 to 84% following HFD, while numbers of CXCR4+ macrophages didn’t change (Fig. 5E). The levels of these receptors in the cells, determined by MFI, showed similar changes to the proportion of macrophages in adipose tissues that expressed these receptors (Fig. 5F). These results indicate that the proportions of macrophages that express chemokine receptors change due to the change of their levels in the cells.

In the PEC, the proportion of macrophages with cell surface expression of CCR2, CCR5, and CXCR4 increased significantly in response to HFD (Fig. 5G). The proportion of CCR2+ expressing macrophages increased 2-fold, CCR5+ macrophages increased 4-fold, whereas CXCR4+ macrophages increased 20-fold. Similarly, levels of these receptors, indicated by MFI, increases with HFD (Fig. 5H). This indicates that the vast majority of macrophages in the PEC do not express markers such as CD206 or CD11c, but express CCR2, CCR5, and CXCR4 with HFD, which may contribute to the “macrophage disappearance.” Therefore, CCR2 increases in microglia and macrophages in the hypothalamus in obesity, while microglia additionally upregulate CXCR4.

### Adipose tissue macrophages can infiltrate the hypothalamus

**Fig. 6:**
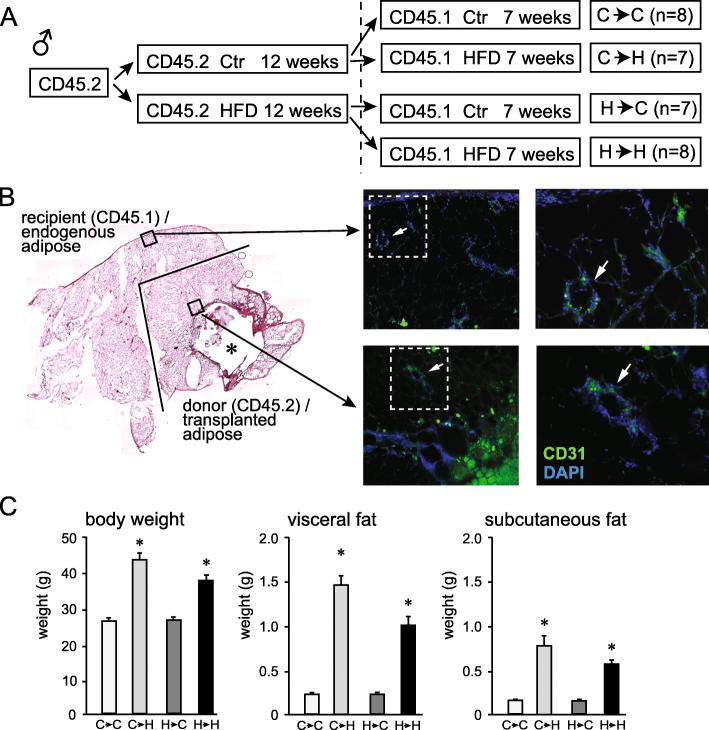
Fat transplant. **A** Experimental design: CD45.2 mice were fed control (Ctr) or high fat diet (HFD) for 12 weeks. Their visceral fat depots were transplanted into visceral depots of 8-week-old recipient CD45.1 mice that were then placed on either Ctr or HFD for additional 7 weeks. The column on the right indicates n in each group and the name of the group, where the first letter indicates the diet of the donor mouse and the second letter shows the diet of the recipient mouse. **B** Visceral fat depot that contains donor adipose tissue (donor) and recipient adipose tissue (endogenous). Left, H&E stain to observe histology. Right side, immunofluorescent staining using CD31 (green) and DAPI (blue) to detect endothelial cells and demonstrate vascularization of transplanted tissue in the bottom panels compared to the endogenous tissue in the top panels (enlarged areas indicated with squares). **C** Body weight, visceral fat depot weight, and subcutaneous fat depot weight. Statistically significant difference (*) between control-fed recipient (second letter **C**) and HFD-fed recipient (second letter **H**) were determined by ANOVA followed by Tukey’s pos thoc test

Our results in Figs. 3 and 4 demonstrated increased numbers of M1-like CD11c+ macrophages in the male hypothalamus following HFD, which also increased in the adipose tissue with HFD. Therefore, to determine whether hypothalamic macrophages are recruited from the periphery, specifically from the visceral adipose tissue, we performed congenic adipose tissue transplant. After 12 weeks on respective diets, visceral fat pads from the donor CD45.2 male mice were isolated and transplanted into the recipient CD45.1 male mice. Since each donor mouse (first letter, C or H, corresponding to control and HFD) had a left and a right fat pad, one was transplanted into the recipient mouse that was placed on the control diet and the other into the recipient mouse that received the HFD for an additional 7 weeks (second letter, C or H, respectively, Fig. 6A). First, we demonstrated viability and vascularization of the transplanted tissue (Fig. 6B). Although there was a necrotic area in the middle of the transplanted tissue, indicated with a star, the majority of tissue was viable and exhibited normal morphology. Immunohistochemical staining for endothelial cell marker, CD31, demonstrated that transplanted fat was vascularized and contained blood vessels (Fig. 6B, bottom images; black square in the H&E stained sections on the left are presented enlarged in fluorescently labeled section on the right), whereas endogenous tissue served as control (Fig. 6B, top images). This is consistent with previous studies, where transplanted fat tissue maintained viability in the recipient adipose tissue [48–50]. Fluorescent staining shows capillaries stained with green CD31, indicated with an arrowhead, in an area distant from the endogenous tissue. This demonstrated that vascularization occurred throughout the transplant, maintaining viability of the transplanted fat tissue. Antibody specificity for CD45.1 and CD45.2 isoforms was confirmed using the visceral fat and spleen from control CD45.1 and CD45.2 mice, without the transplant (data not shown). As expected, animals fed HFD after transplant were heavier than animals fed control diet, regardless of the donor diet (Fig. 6C). Both visceral fat pad, which received the transplant, and subcutaneous fat pad, which is distant and physiologically different, increased in size and weight following 7-week exposure to HFD (middle and right graphs in Fig. 6C). The transplanted fat pad from a donor on control diet to the recipient that was fed HFD (C→H) gained more weight compared to the transplanted fat pad from a donor on control diet followed by recipient on control diet (C→C). Similarly, donor fed HFD transplanted to the recipient fed HFD (H→H) gained more weight than donor fed HFD transplanted to control diet recipient (H→C), also indicating survival and vascularization, and excess fat deposition (Fig. 6C, middle graph). Similar results were obtained analyzing the subcutaneous fat depot. Interestingly, weight gain of H→H was lower than C→H (less weight gain was observed between H→C and H→H than between C→C and C→H), when comparing body weight, visceral fat depots and subcutaneous fat depots. This may imply that transplanted tissue from HFD fed animals sends signals to the recipient that limits the growth of the fat depot and body weight gain, as postulated in previous studies [49, 50].

**Fig. 7:**
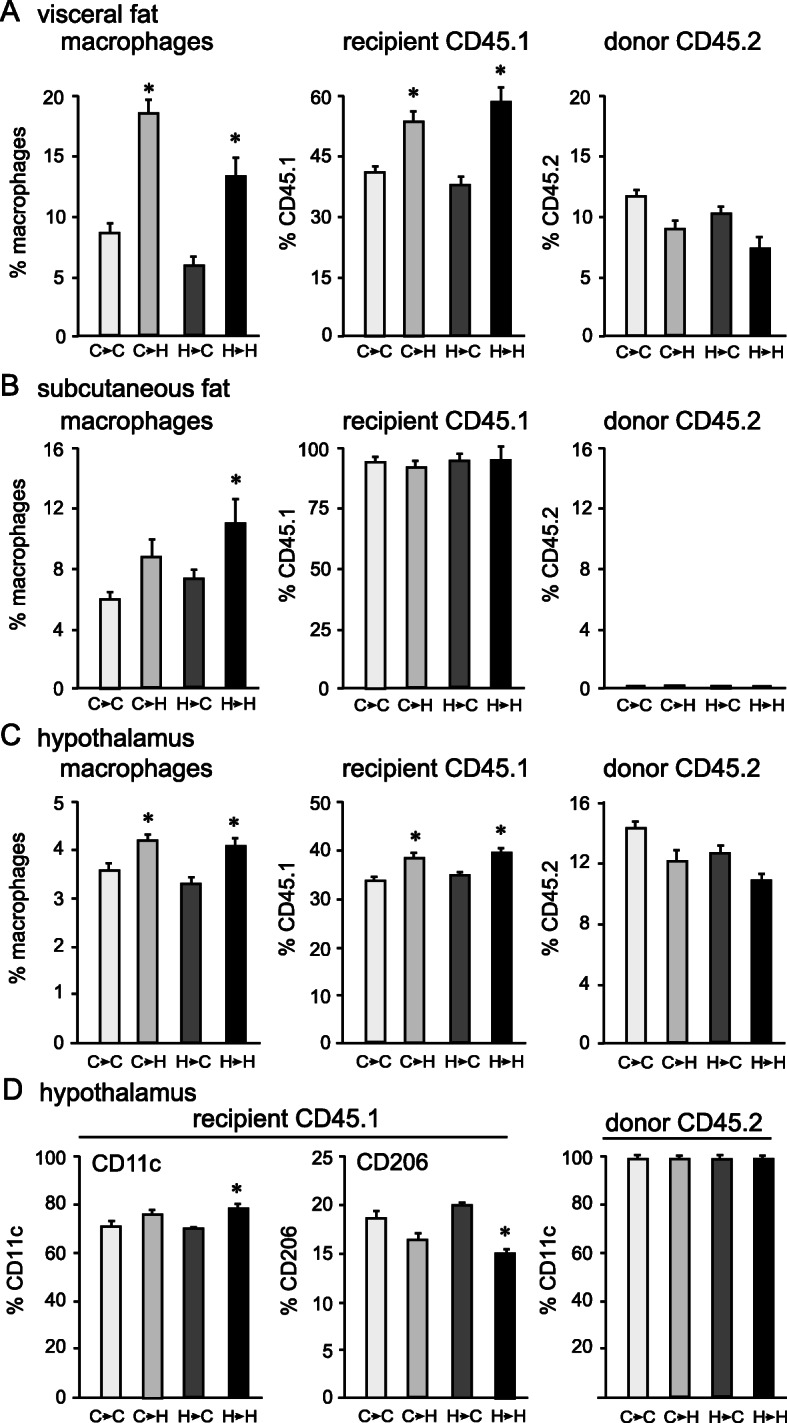
Macrophages migrate from visceral fat pad to the hypothalamus. **A** Visceral fat pads that contained both donor and endogenous tissue show increase in macrophage percentage following HFD compared to control diet, regardless of donor diet. **B** Subcutaneous fat depot only contains recipient endogenous CD45.1, indicating that macrophages do not infiltrate subcutaneous fat depot. **C** Hypothalamus demonstrates increase in macrophage proportion following HFD (left panel), presence of endogenous, recipient CD45.1 (middle panel) and donor CD45.2 macrophages (right panel). **D** Proportion of pro-inflammatory CD11c+ macrophages and resident CD206+ macrophages within CD45.1 and CD45.2 hypothalamic macrophages. Statistical significance (*) between control-fed recipient (second letter **C**) and HFD-fed recipient (second letter **H**) were determined by ANOVA followed by Tukey’s post hoc test

From these mice (n indicated in Fig. 6A), tissues were analyzed by flow cytometry to detect donor (CD45.2) and recipient (CD45.1) macrophages. The visceral fat pad (Fig. 7A), the subcutaneous fat pad (Fig. 7B), and the hypothalamus (Fig. 7C), were collected from recipient CD45.1 mice and examined for the presence of CD45.2 donor macrophages. The percent of total macrophages (both recipient CD45.1 and donor CD45.2) in the visceral fat pad from the HFD-fed mice was higher than control-fed mice, similarly to previous results from Fig. 3. The visceral adipose depot from C→H mice contained a larger proportion of macrophages compared to C→C, and H→H had more macrophages than H→C (Fig. 7A, left panel), indicating that HFD exposure increases the accumulation of macrophages into the fat. Not surprisingly, endogenous, recipient CD45.1 macrophages were more abundant in mice fed HFD regardless of the donor diet: C→H had more CD45.1 macrophages than C→C, and H→H had more than H→C (Fig. 7B, middle panel). Significantly, donor CD45.2 macrophages were present in the fat pad regardless of the diet, indicating that donor cells survive 7 weeks post-transplant.

The subcutaneous fat depot served as a control, since similarly to the hypothalamus, this depot did not receive the transplanted tissue but was exposed to control and HFD diet. Subcutaneous adipose tissue is less inflammatory in obesity [50]; thus, the accumulation of macrophages was lower, and the difference between C→H compared to C→C was smaller than in the visceral depot (Fig. 7B, left panel). All of the macrophages in the subcutaneous fat pad were endogenous recipient CD45.1 macrophages (Fig. 7B, middle panel). Significantly, the subcutaneous fat depot from any group lacked donor CD45.2 macrophages, indicating that macrophages from the visceral fat do not migrate to the subcutaneous fat depot.

Ultimately, hypothalami were analyzed for the presence of donor CD45.2 macrophages that were introduced into the CD45.1 mouse with a transplanted adipose tissue. Hypothalami from mice on the HFD after transplant, accumulated macrophages and exhibited an increase in the number of total macrophages compared to mice on control diet, regardless of the donor diet (Fig. 7C, left panel). The difference between HFD and control was lower than what we observed in Fig. 3, likely corresponding to the shorter duration of the diet after transplant, 7 weeks versus 14 weeks in Fig. 3. Regardless, the differences between HFD and control mice are significant. There was no difference between C→C (3.6%) and H→C (3.2%), or between C→H (4.3%) and H→H (4.1%), although H→C and H→H received transplant from HFD-fed mice, while C→C and C→H received transplant from mice fed control diet. The majority of the macrophages in the hypothalamus were endogenous CD45.1-positive from the recipient mouse tissues: 34% in C→C, 39% in C→H, 35% in H→C, and 40% in H→H. Mice fed HFD after transplant had higher percentage of CD45.1 than mice fed control diet (Fig. 7C middle panel). Importantly, hypothalami contained donor CD45.2 macrophages: 14.4% of macrophages in C→C were CD45.2, 12% in C→H, 12.4% in H→C, and 10.7% in H→H (Fig. 7C, right panel). Since only visceral adipose tissue from the CD45.2 mice was transplanted to the recipient CD45.1 mice, detection of CD45.2 macrophages in the hypothalamus indicates that the macrophages from the donor visceral adipose tissue infiltrate the hypothalamus.

We analyzed the phenotype of hypothalamus-infiltrated macrophages, using pro-inflammatory M1 marker CD11c and anti-inflammatory M2 marker CD206. In the hypothalamus, endogenous CD45.1 CD11c+ pro-inflammatory macrophages comprised a larger proportion than CD45.1 CD206+ anti-inflammatory macrophages (~70% compared to ~20%, compare left and middle panels in Fig. 7D). The increase in CD11c+ macrophages following HFD, from 70% in H→C to 77% in H→H, corresponded to the decrease in CD206+ macrophages from 20% in H→C to 15% in H→H (Fig. 7D), indicating a further shift to the pro-inflammatory phenotype. Donor CD45.2 macrophages, which originated from the transplanted visceral adipose tissue, were all pro-inflammatory CD11c+, and all lacked CD206 (Fig. 7D, right panel). These results indicate that hypothalami are infiltrated by the pro-inflammatory macrophages from the transplanted donor fat depot.

## Discussion

In this study, we demonstrate that HFD-fed male mice have increased infiltration of peripheral macrophages into the hypothalami from the visceral adipose tissues. Macrophage infiltration occurs in addition to microgliosis, which is evidenced by an increase in the number of microglia, resident immune cells of the brain. We further show that HFD induces expression of neuroinflammatory genes in a region-specific manner, which may be defined by macrophage infiltration through the fenestrated capillaries. Peripheral macrophages that infiltrate the hypothalami of obese male mice, have increased surface expression of the chemokine receptor CCR2, which regulates monocyte chemotaxis and recruitment, and an increase in pro-inflammatory markers, CD11c and PDL1. These are the same markers that increase in the adipose tissue macrophages in response to HFD. This prompted us to examine if macrophages can migrate from visceral adipose tissue depots to the hypothalamus. To address this, we performed fat transplant experiments, which revealed that donor macrophages from the transplanted fat tissue are able to migrate into the hypothalamus. Peripheral macrophage infiltration may have profound consequences, causing obesity-mediated hypothalamic neuroinflammation that leads to synaptic plasticity and physiological changes, which we and others have previously reported.

Adipose tissue transplant demonstrates that macrophages from the visceral fat tissue can infiltrate the hypothalamus. Visceral fat contains more infiltrating macrophages and higher expression of inflammatory cytokines than subcutaneous fat [23, 74]. This may explain why transplanted macrophages do not infiltrate the subcutaneous adipose tissue that is partially resistant to inflammatory changes in obesity. We postulated that macrophages activated in the visceral adipose tissue infiltrate the hypothalamus, since they can infiltrate liver and muscle and contribute to insulin resistance [37–40]. Macrophage infiltration occurs primarily in male mice. We and others determined differences in fat deposition between males and females [9, 75–77]. Normal weight human males and male mice fed a normal diet have larger visceral fat depots than females, when normalized to the total body weight. After exposure to the high fat diet, males deposit fat into the visceral depot, while females preferentially deposit fat into the subcutaneous depot, exacerbating the difference [9, 78]. Since the visceral depot is more metabolically active and more inflammatory [50, 79], differences in fat deposition could contribute to sex differences in inflammation and explain why males accumulate more macrophages into adipose depots, which once activated infiltrate other tissues. Thus, differences in fat depots, and not absolute adiposity, may impact obesity-induced inflammation and macrophage activation [75, 76, 80].

Prior studies analyzing effects of obesity reported that neuroinflammation was specific for the arcuate nucleus of the hypothalamus [81, 82]. Since the arcuate nucleus contains neurons that regulate metabolism and energy expenditure, it was hypothesized that neuroinflammation occurs in response to neuronal stress. Our previous studies detected neuroinflammatory changes in the medial preoptic area (POA), in addition to the arcuate nucleus [9]. The POA is located next to the OVLT, while the arcuate nucleus is juxtaposed to the median eminence, both areas of the hypothalamus that contain fenestrated capillaries and a leaky blood-brain barrier. Although the brain has long been regarded as an immune-privileged site due to the blood-brain barrier, hypothalamic areas that contain fenestrated capillaries allow the hypothalamus to respond to metabolic and inflammatory changes in the periphery [20]. Here, we demonstrate additional regional differences in gene expression in HFD, using NanoString analyses. Compared to the anterior portion that encompasses fenestrated capillaries in the OVLT, the posterior portion that contains fenestrated capillaries in the median eminence demonstrated more changes in HFD-induced gene expression. This may reflect the greater vascular permeability of the median eminence [51, 52]. Therefore, we posit that the leaky blood-brain barrier allows macrophages to infiltrate the hypothalamus, and further postulate that location, rather than neuronal function, is an important determinant of impaired hypothalamic functions driven by obesity-mediated neuroinflammation.

Macrophages are arguably the main immune contributors to obesity-mediated chronic inflammation, since they accumulate in the adipose tissues and after activation, infiltrate other tissues in obesity [39, 40, 83]. Within the brain, recruited macrophages are difficult to distinguish from resident microglia by immunostaining. Therefore, we analyzed these populations by flow cytometry and determined that following HFD, infiltration of peripheral, circulatory macrophages occurs in addition to increased numbers of microglia. Thus, both cell populations can contribute to the neuroinflammation induced by obesity. However, it is still not clear what occurs first. Microglia may sense changes in metabolic markers and become activated, which causes influx of peripheral macrophages [84]. Alternatively, macrophages that are activated in response to enlarged adipose tissue mass, accumulation of reactive oxygen species, and increased fatty acids concentration [85, 86], can infiltrate the hypothalamus, as occurs in the liver and the muscle [37–40]. Using fat transplantation, we demonstrate that macrophages from visceral adipose tissue can infiltrate the hypothalamus, and future studies will determine if this occurs prior to microglia activation. These neuroinflammatory changes caused by peripheral macrophages likely contribute to the synaptic plasticity that we and others demonstrated previously [9, 43], which leads to changes in neuron connectivity and therefore their function.

Cytokines and chemokines that contribute to chemotaxis to the hypothalamus have not been previously elucidated. CCL2 / MCP-1 is elevated in obesity [23] and contributes to insulin resistance, hepatic steatosis, and macrophage accumulation in adipose tissue [26, 87]. Here, we show that hypothalamic macrophages have increased surface levels of the receptor for CCL2, CCR2, which may contribute to macrophage accumulation to the hypothalamus. The proportion of microglia that express CCR2 also increases in obesity, which may indicate their activation, or chemoattraction toward areas with fenestrated capillaries and the gradient of increased leptin, free fatty acids, or insulin. A previous study demonstrated that expression of CCL5 and its receptor, CCR5, increases in adipose tissue following HFD [31]. Consistent with this study, we observed that adipose tissue macrophages increase CCR5 levels following HFD, which may contribute to macrophage accumulation to the adipose tissue. However, we did not observe an increase in CCR5+ macrophages in the hypothalamus. Since CCR5 is already present in the majority of macrophages within the hypothalamus, it is possible that CCR5 is a characteristic of hypothalamic macrophages regardless of diet. CXCR4 in the brain is critical during development for neurogenesis, migration, and angiogenesis. In adults, it modulates neuronal function via interaction with neurotransmitter receptors [36, 88]. In the periphery, CXCR4 has different functions in immune cell migration depending on its ligand. Although the percentage of CXCR4-positive macrophages did not change with HFD in our studies, the percentage of CXCR4+ microglia increased. This may implicate microglia in modulation of neurotransmitter receptor levels and synaptic plasticity in obesity.

Macrophages are the main cell population in the peritoneum and represent the first line of defense against infection [66, 67]. Peritoneal macrophages can be recruited to other tissues: for example, they can rapidly migrate to the liver in response to liver injury [89, 90]. Consistent with other studies [65, 67], we observed that HFD led to lower percentage of macrophages in the peritoneum, decreased peritoneal macrophage expression of the M2 marker, CD206, and increased chemokine receptors levels. Our results may indicate that the peritoneal cavity serves as a depot from which macrophages are recruited to other tissues, including adipose tissue, similarly to their recruitment to the liver following injury.

Obesity-associated diseases exhibit significant sex differences [44, 45]. Sex steroid hormones and intrinsic sex differences in the immune system due to chromosomal differences may contribute to these sex-specific responses in diet-induced obesity [78, 91–94]. We previously demonstrated that females may be protected due to the higher production of anti-inflammatory cytokines such as IL-10 in the hypothalamus, adipose tissue, and by macrophages themselves [9, 28]. Herein, we demonstrate sex-specific changes in hypothalamic inflammation, using gene expression analyses, and identify several other candidates with roles in metabolism and energy expenditure that may provide protection to females. Only male hypothalami, adipose tissues and PECs exhibit changes in macrophage numbers in obesity, which may correlate with male-specific enhancement of myelopoiesis [94] or the higher migratory capacity of male macrophages [28]. Since we posit that adipose tissue is a source of hypothalamic macrophages in obesity, differential fat deposition, as discussed above, may explain some of these observations. Therefore, differential fat deposition likely causes larger accumulation of macrophages in males, which have macrophages that are intrinsically more inflammatory and more migratory than females [28]. This in turn, causes adipose tissue-activated macrophages to migrate to other tissues such as the liver, muscle, or hypothalamus, but not the subcutaneous fat tissue. Tissue selectivity may depend on the vasculature permeability, which future studies will address. Macrophage influx in the liver and the hypothalamus may contribute to male-biased propensity for obesity-induced pathologies such as heart disease and the metabolic syndrome.

## Conclusion

In these studies, we identify a connection between macrophage accumulation in the visceral adipose tissue and hypothalamic neuroinflammation in response to obesity. Since males fed high fat diet specifically demonstrate accumulation of macrophages in all tissues examined, we analyzed male macrophages in more detail and determined that male macrophages in the hypothalamus had increased levels of pro-inflammatory markers and markers of metabolically activated macrophages. Since the same markers are increased in adipose tissue macrophages, we postulated that adipose tissue may be a source of hypothalamic macrophages that accumulate in obesity. Using adipose tissue transplantation, we demonstrate that macrophages from visceral adipose tissue infiltrate the hypothalamus. Our studies reveal a new association between the adipose tissue and the central nervous system and may explain obesity-induced impairments in the hypothalamic function.

## Data Availability

All data generated or analyzed during this study are included in this published article or in supplemental materials.

## Abbreviations

BMI: Body mass index
HFD: High fat diet
OVLT: Organum vasculosum laminae terminalis
PEC: Peritoneal cavity
MCP-1: Monocyte chemoattractant protein-1
POMC: Pro-opiomelanocortin
NPY: Neuropeptide Y
AgRP: Agouti-related protein
TRH: Thyrotropin-releasing hormone
MC4R: Melanocortin 4 receptor
ATM: Adipose tissue macrophages
SDF-1: Stromal derived growth factor
MFI: Mean fluorescent intensity
PDL-1: Programmed death-ligand 1
